# Predictors of breastfeeding among women in Lao People’s Democratic Republic: a prospective cohort study

**DOI:** 10.3389/fgwh.2026.1663003

**Published:** 2026-06-03

**Authors:** Kianna Dufault, Jordyn T. Wallenborn

**Affiliations:** 1 Department of Public Health Fargo, North Dakota State University, North Dakota, United States; 2 Department of Epidemiology and Public Health, Swiss Tropical and Public Health Institute, University of Basel, Basel, Switzerland

**Keywords:** breastfeeding predictors, breastfeeding, Lao PDR, pregnancy, women’s health

## Abstract

Breastfeeding is a worldwide practice that provides many health benefits for both mothers and babies. However, many women still do not breastfeed for the recommended duration, or even at all. Lao People’s Democratic Republic (Lao PDR), a country in Southeast Asia, has very low breastfeeding rates compared with neighboring countries. We conducted a prospective cohort study to identify predictors of breastfeeding among women in Lao PDR. Participants in this study were drawn from a multigenerational birth cohort that was launched in Vientiane Capital, Lao PDR, in 2020. A survey was conducted at approximately 1 month postpartum, which included both breastfeeding and non-breastfeeding women. In unadjusted analyses, we found that maternal age, education level, and occupation were associated with breastfeeding status at 30 days postpartum. After adjustment, occupation, daily physical activity, joint pain, and exclusive breastfeeding intention were the metrics associated with breastfeeding status. These findings suggest that prenatal intention and maternal health characteristics are associated with breastfeeding continuation in Lao PDR. The results emphasize the importance of further research to identify breastfeeding practices.

## Introduction

Breastfeeding is an important practice with many health benefits for both infants and mothers. Infants who are breastfed have a lower risk of asthma, obesity, type 1 diabetes, sudden infant death syndrome (SIDS), otitis, and viral gastroenteritis. Mothers who breastfeed are at reduced risk of developing cancer, type 2 diabetes, and high blood pressure (1). Improving breastfeeding rates could prevent 823,000 annual deaths in children under five and 20,000 annual deaths from breast cancer (2). Along with preventing deaths in children and mothers, if optimal breastfeeding is achieved, it could reduce global healthcare costs by approximately 300 billion United States Dollars (USD) (3).

**Figure 1:**
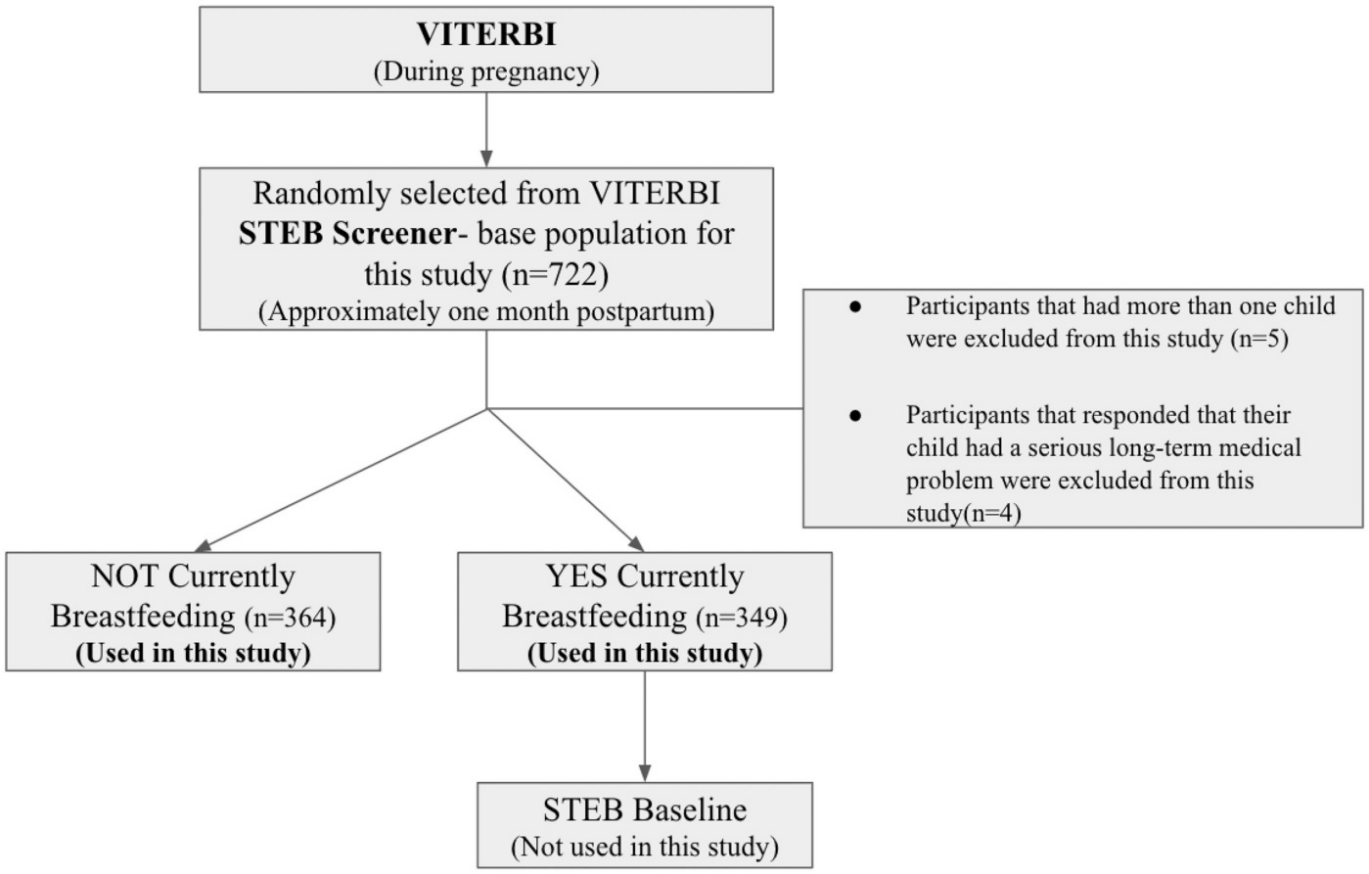
Base population flowchart showing inclusion and exclusion criteria.

Breastfeeding rates remain suboptimal, with only 48% of infants under 6 months of age worldwide breastfed exclusively as of 2023. South Asia has the highest percentage of infants aged under 6 months exclusively breastfed, at 60% (4). However, an outlier in the region is Lao People’s Democratic Republic (Lao PDR). As of 2023, only 50.3% of children under 6 months were exclusively breastfed in Lao PDR (5). Despite Vientiane being the largest city and capital of Lao PDR, it has one of the lowest exclusive breastfeeding rates in the country, with only 33.7% of children aged 0–5 months being exclusively breastfed (5). Lao PDR faces multiple challenges as a low-income country with low literacy and numeracy rates, malnutrition, lack of infrastructure, natural disasters, and contamination due to explosive remnants of war, which restrict the amount of farming that citizens can do, contributing to a lack of food (6). Investigating what makes Vientiane an outlier compared with the rest of the country is an important step in increasing breastfeeding rates for the city and Lao PDR overall. Data analyses to identify predictors of breastfeeding postpartum can help provide more information for policy decisions, as current literature identifying factors influencing breastfeeding in Lao PDR is limited.

It is recommended by the World Health Organization (WHO), United Nations Children's Fund (UNICEF), and the 2020–2025 Dietary Guidelines for Americans that infants be exclusively breastfed for the first 6 months of life (7). Although breastfeeding is the ideal source of infant nutrition, research shows that some women are not able to produce enough breast milk (8). Women who struggle most with insufficient milk supply are those with insulin resistance, prediabetes, or type 2 diabetes (9). Other reasons why women stop breastfeeding include inconvenience and fatigue associated with breastfeeding (10).

Evidence on determinants of breastfeeding in Lao PDR is limited, but studies from Southeast Asia and other low- and middle-income countries suggest that maternal employment, urbanization, health system support, and social norms may be important influences on early breastfeeding practices (11). Identifying predictors associated with early breastfeeding continuation in Lao PDR is therefore critical to advancing maternal and child health. A prospective cohort study was conducted to identify prenatal and maternal predictors of breastfeeding at 30 days postpartum in Vientiane Capital. Lower breastfeeding rates in Lao PDR contribute to the challenges that it faces by increasing the risk of poor maternal and child health outcomes (12).

## Methods

### Study design and setting

This study is a secondary data analysis using information drawn from two different sources. The first source was the Vientiane Multigenerational Birth Cohort (VITERBI), a multigenerational birth cohort that was launched in Vientiane Capital in 2020, with enrollment continuing until late 2023 (13). VITERBI enrolls pregnant women during their first or second trimester, along with the child's family. Two interviews are conducted: one during pregnancy to gather sociodemographic data, along with physical data such as blood pressure and anemia levels, and another 2–8 weeks after the birth of the child to identify pregnancy outcomes (14). The goal of VITERBI was to identify gaps and health challenges in Lao PDR to develop programs to improve population health in the future (14).

Women who had given birth in the previous four weeks were randomly selected from VITERBI and screened for inclusion in the Social Transfers for Exclusive Breastfeeding (STEB) randomized controlled trial (15), which constituted the second source. Participants were enrolled individually. No cluster-based sampling or district-level randomization was used. The screener STEB survey was taken approximately 1 month postpartum, resulting in a sample size of 722 participants. Out of those, nine women were excluded, which resulted in a sample size of 713. In the screener survey, participants were asked about their recent birth and breastfeeding behaviors. Inclusion criteria were completion of the ∼1-month postpartum screener. Exclusion criteria included multiple births and/or infants with serious long-term medical problems. These women were asked the following: “Does your baby have any serious, long-term medical problems?” “Did you have twins or more than one baby?” Participants who reported more than one child were excluded from the study (*n* = 5), along with participants whose infants had a serious long-term medical problem (*n* = 4). The primary outcome for this analysis was current breastfeeding status (any vs. none) ∼30 days postpartum. Participants answered yes (*n* = 349) or no (*n* = 364) when asked (Figure 1).

### Ethical consideration

This study was submitted for approval to the National Ethics Committee for Health Research of the Ministry of Health (MoH) in Vientiane, Lao PDR and the Ethical Committee of Northwest and Central Switzerland. All protocol amendments and informed consent procedures were reviewed and approved by an Independent Ethics Committee (IEC).

### Potential confounders

Variables included in the multivariable model were selected based on conceptual relevance. Maternal age and occupation were included as key sociodemographic factors. Prenatal intention to exclusively breastfeed and maternal health characteristics such as daily physical activity and joint pain were examined as well.

### Study variables

#### Outcome variable

The primary outcome variable was breastfeeding status at ∼30 days postpartum. Breastfeeding status was based on the self-reported response to the survey question “Are you currently breastfeeding?” (yes/no). For regression analyses, current breastfeeding (yes) was coded as the outcome event.

#### Predictor variables

There are many different metrics that can impact breastfeeding status. The metrics analyzed were health issues, work status, support network, and home life. Within these metrics, there are many different variables. Health issues include variables like pain, arthritis, and mental health complications. Work status involves variables such as income and employment status. Support network includes satisfaction with support from friends and family and attending a health promotion session where breastfeeding was discussed.

### Data analysis

Descriptive statistics are presented in Table 1 as frequencies and percentages. Bivariate associations between predictors and breastfeeding status were assessed using chi-square tests or Fisher's exact tests when the cell counts were small. Multivariable logistic regression models were constructed to examine factors independently associated with current breastfeeding at 1 month postpartum. Analyses were conducted using complete case analysis. Odds ratios (ORs) and 95% confidence intervals (CIs) are reported, with statistical significance defined as *p* < 0.05. All analyses were performed using R (packages: tidyverse, sjPlot).

**Table 1:** Participant characteristics by breastfeeding status at ∼30 days postpartum (*n* = 713).

Predictor	Total, *n* (%)	Breastfeeding Yes *n* (%)	Breastfeeding No *n* (%)	*p*-Value
Age				**0**.**04**
≤19	88 (12.3)	32 (36.4)	56 (63.6)	
20–29	399 (56.0)	199 (49.9)	200 (50.1)	
≥30	226 (31.7)	118 (52.2)	108 (47.8)	
Marital status				0.32
Never married	55 (7.7)	29 (52.7)	26 (47.3)	
Currently married	630 (88.4)	310 (49.2)	320 (50.8)	
Separated/divorced/widowed/cohabitating/refused	28 (3.9)	10 (35.7)	18 (64.3)	
Education level				**0**.**048**^a^
No schooling/primary	155 (21.8)	89 (57.4)	66 (42.6)	
Secondary	322 (45.2)	142 (44.1)	180 (55.9)	
University	226 (31.7)	113 (50.0)	113 (50.0)	
Higher	9 (1.3)	5 (55.6)	4 (44.4)	
Work status				0.71
Government employee	50 (7.0)	25 (50.0)	25 (50.0)	
Non-government employee	103 (14.4)	50 (48.5)	53 (51.5)	
Self-employed	178 (25.0)	82 (46.1)	96 (53.9)	
Non-paid	14 (2.0)	4 (28.6)	10 (71.4)	
Student/unemployed	18 (2.5)	10 (55.6)	8 (44.4)	
Homemaker	184 (25.8)	94 (51.1)	90 (48.9)	
Other	166 (23.3)	84 (50.6)	82 (49.4)	
Occupation^b^				**0**.**02**
Farmer	69 (12.2)	44 (63.8)	25 (36.2)	
Laborer	16 (2.8)	7 (43.8)	9 (56.3)	
Self-employed	57 (10.1)	22 (38.6)	35 (61.4)	
Employee with salary	143 (25.3)	77 (53.8)	66 (46.2)	
Merchant	158 (28.0)	66 (41.8)	92 (58.2)	
Housewife	50 (8.8)	32 (64.0)	18 (36.0)	
Other	72 (12.7)	36 (50.0)	36 (50.0)	
Monthly income (Laotian Kip)^c^				0.74
≤1,500,000	260 (36.5)	132 (50.8)	128 (49.2)	
≤3,500,000	212 (29.7)	100 (47.2)	112 (52.8)	
≥3,500,000	93 (13.0)	46 (49.5)	47 (50.5)	

a Fisher's exact test.

b Occupation data missing for 148 participants (20.8%) *n* = 565.

c Monthly income data missing for 148 participants (20.8%) *n* = 565.

*P*-values were calculated using chi-square or Fisher's exact test as appropriate. Percentages are row percentages. Occupation and monthly income data missing for 148 participants.

Bold values are values that were found to be statistically significant.

## Results

### Breastfeeding practices

The sample size of this prospective cohort study was 713 women, of which 349 (48.9%) were currently breastfeeding and 364 (51.1%) were not at the time of survey administered. Most of the participants fell within the age range of 20–29 years (56.0%), while 31.7% were 30 years or older and 12.3% of participants were 19 years or younger. Most participants were currently married (88.4%), while 7.7% were never married and 3.9% were separated, divorced, widowed, cohabitating, or refused to answer. As far as work status, most of the participants were homemakers (25.8%) or self-employed (25%). With regard to monthly income in Laotian Kip, 36.5% made ₭1,500,000 or less, 29.7% made ₭3,500,000 or less, and 13.0% made the highest monthly income of greater than ₭3,500,000 (Table 1).

In unadjusted analyses, maternal age and occupation were associated with breastfeeding status. In multivariable logistic regression models, older maternal age, occupation, daily physical activity, joint pain in the past 12 months, and prenatal intention to exclusively breastfeed were independently associated with current breastfeeding at 30 days postpartum (Table 2).

**Table 2:** Sociodemographic factors with odds ratios and 95% confidence intervals.

Predictor	Crude odds ratio (COR)	95% CI	Adjusted odds ratio (AOR)	95% CI
Age				
≤19	Reference	Reference	Reference	Reference
20–29	1.52	0.85–2.79	1.77	0.96–3.33
≥30	1.56	0.84–2.92	1.73	0.91–3.34
Occupation				
Farmer	1.57	0.88–2.83	1.31	0.71–2.45
Laborer	1.2	0.42–3.52	2.59	0.58–12.16
Self-employed	0.67	0.36–1.25	0.69	0.36–1.13
Employee with salary	Reference	Reference	Reference	Reference
Merchant	0.77	0.49–1.21	0.79	0.49–1.26
Housewife	1.91	0.99–3.76	**2.15**	**1.09–4.43**
Other	1.42	0.80–2.52	1.38	0.77–2.49
Physical activity				
Yes	Reference	Reference	Reference	Reference
No	**0.55**	**0.36–0.85**	**0.56**	**0.35–0.90**
Joint pain				
Yes	Reference	Reference	Reference	Reference
No	**0.34**	**0.13–0.79**	**0.38**	**0.14–0.90**
Feeding plan				
Not exclusive	Reference	Reference	Reference	Reference
Exclusive	**1.87**	**1.16–3.06**	**1.77**	**1.08–2.95**

Crude and adjusted models were restricted to participants with complete data on included covariates (*n* = 565). Odds ratios represent odds of breastfeeding at approximately 30 days postpartum.

Bold values are values that were found to be statistically significant.

Women who reported not having 30 min of daily physical activity at work and/or during leisure time had lower odds of breastfeeding. Prenatal intention to exclusively breastfeed was associated with higher odds of breastfeeding at 1 month postpartum.

### Sociodemographic characteristics of the participants

Bivariate analyses revealed significant associations between breastfeeding status at 30 days postpartum and maternal age (*p* = 0.04) and occupation (*p* = 0.02) (Table 1). We found that older women were more likely to report current breastfeeding at 1 month postpartum compared with those 19 years or younger. In unadjusted analyses, education level was marginally associated with breastfeeding status. Occupation was shown to be associated with breastfeeding status, with differences between categories of occupation.

Table 2 presents crude and adjusted odds ratios for factors including maternal age, occupation, daily physical activity, joint pain in the past 12 months, and prenatal intention to exclusively breastfeed. Several factors were found to be associated with breastfeeding status. In adjusted models, women aged 20–29 years and 30 years or older had higher odds of breastfeeding compared with women aged 19 years or younger. Occupation remained associated with breastfeeding status, with homemakers showing higher odds of breastfeeding compared with salaried employees. Women reporting no daily physical activity had lower odds of breastfeeding. Prenatal intention to exclusively breastfeed was associated with higher odds of breastfeeding at 1 month postpartum. Joint pain in the past 12 months was also associated with breastfeeding status in adjusted analyses.

## Discussion

In our analysis, we found that maternal age, occupation, physical activity, joint pain, and prenatal intention to exclusively breastfeed were metrics associated with breastfeeding status at 30 days postpartum.

Older maternal age was associated with higher odds of breastfeeding compared with younger maternal age (19 years and younger). In other published literature, age has been shown to be a factor in breastfeeding status, but there is no strong evidence as to why. One study points out that although there is no strong evidence, infant difficulties such as latching and sucking problems are often cited by younger mothers as the reason for stopping breastfeeding. Along with this, differences in infant characteristics suggest that initiation of breastfeeding can be stressful for some mothers and infants (16).

Another metric we found to be significant in our analysis was occupation. Occupation influences breastfeeding status for a variety of reasons, with the main reason being that homemakers can feed on demand but employees must step out of work to breastfeed. A cross-sectional study in the Kingdom of Saudi Arabia showed that all non-working women breastfed their babies, and 92% of working women breastfed, while the remaining 8% of working women did not breastfeed. The results also showed that only 7% of working women practiced exclusive breastfeeding, while 37% of non-working women exclusively breastfed (17). This is likely due to the availability of non-working women to breastfeed compared with working women. These findings support our analysis, which shows that homemakers demonstrated higher odds of breastfeeding compared with salaried employees (AOR = 2.15, 95% CI 1.09–4.43).

Physical activity also plays an important role in breastfeeding status. A similar prospective cohort study of 2,030 Vietnamese women found that higher levels of physical activity by pregnant women were associated with improved breastfeeding outcomes (18). Physical activity has not only been shown to improve breastfeeding outcomes but also has many benefits for women. While the causal direction cannot be determined, being physically active and breastfeeding can also support a new mother's physiological and psychological health (19). These findings from similar studies support our results that physical activity is positively associated with breastfeeding status in adjusted analyses.

We also found that joint pain in the past 12 months was associated with breastfeeding status at 30 days postpartum. The reason for this is unclear; however, joint pain is also a symptom of depression (20). Depression can play a role in breastfeeding duration as mothers with depressive symptoms were found to be less likely to continue breastfeeding through 2–4 months postpartum compared with mothers without depressive symptoms (21). However, because joint pain was measured retrospectively and breastfeeding status was assessed postpartum, temporal direction cannot be established.

Our analysis found that prenatal intention to exclusively breastfeed was independently associated with breastfeeding at 1 month postpartum. A cohort study done in Lebanon and Qatar in 2022 showed that both a positive attitude toward breastfeeding and strong intention to breastfeed were independent predictors of exclusive breastfeeding at 4 months, as well as breastfeeding at 4 and 6 months (22).

## Limitations and strengths

Several limitations should be considered when interpreting these results. First, breastfeeding status was self-reported, which may introduce recall or reporting bias. Second, since this was a secondary data analysis, the study was not powered to detect associates for all predictors. Third, there was no question in the survey on breastfeeding initiation. This is a limitation because we do not know if women who said they were not currently breastfeeding ever attempted to breastfeed or for how long. Lastly, we had missing data, which resulted in reduced sample size for multivariable analyses due to listwise deletion, which may introduce bias if missingness was not completely random.

However, this study has some notable strengths. Both women who were currently breastfeeding and those not currently breastfeeding were included in the study, which allowed for direct comparison. As data were collected prospectively through an established cohort, we were able to look at multiple different metrics of breastfeeding, both prenatally and postpartum, which was beneficial in strengthening the temporal interpretation for prenatal exposures such as breastfeeding intention.

## Conclusion

In this prospective cohort study of women in Vientiane Capital, Lao PDR, maternal age, occupation, physical activity, joint pain, and prenatal intention to exclusively breastfeed were associated with current breastfeeding at approximately 30 days postpartum. Based on our research from this study, we recommend that younger women be targeted, particularly those that are less than 19 years of age, since this is the age group that had the poorest breastfeeding outcomes. We also recommend that pregnant women and women who have just given birth are encouraged by medical professionals to practice physical activity since it has many benefits for physiological and psychological health (23). Attending a health education session where breastfeeding was discussed was also shown to be beneficial in terms of breastfeeding outcomes. Lastly, we recommend that further research be conducted regarding occupation of new mothers to determine the best possible breastfeeding outcome for working mothers. However, given the observational nature of this study, findings should be interpreted as associations rather than causal effects.

## Data Availability

The data analyzed in this study are subject to the following licenses/restrictions: N/A. Requests to access these datasets should be directed to Dr. Jordyn Wallenborn (jordyn.wallenborn@swisstph.ch).
